# Fatal Occupational Asthma in Cannabis Production — Massachusetts, 2022

**DOI:** 10.15585/mmwr.mm7246a2

**Published:** 2023-11-17

**Authors:** Virginia M. Weaver, Jeremy T. Hua, Kathleen M. Fitzsimmons, James R. Laing, Wigdan Farah, Anne Hart, Trapper J. Braegger, Michelle Reid, David N. Weissman

**Affiliations:** ^1^Office of Occupational Medicine and Nursing, Directorate of Technical Support and Emergency Management, Occupational Safety and Health Administration, U.S. Department of Labor, Washington, DC; ^2^Division of Environmental & Occupational Health Sciences, National Jewish Health, Denver, Colorado; ^3^Occupational Health Surveillance Program, Massachusetts Department of Public Health; ^4^Division of Pulmonary and Critical Care Medicine, Mayo Clinic, Rochester, Minnesota; ^5^Region 1, Occupational Safety and Health Administration, U.S. Department of Labor, Springfield, Massachusetts; ^6^Salt Lake Technical Center, Directorate of Technical Support and Emergency Management, Occupational Safety and Health Administration, U.S. Department of Labor, Salt Lake City, Utah; ^7^Respiratory Health Division, National Institute for Occupational Safety and Health, CDC.

**Summary**

**What is already known about this topic?**

Occupational allergic diseases, including asthma, are an emerging concern in the rapidly expanding U.S. cannabis industry.

**What is added by this report?**

In 2022, the first death attributed to occupational asthma in a U.S. cannabis production worker occurred in Massachusetts. This case illustrates missed opportunities for prevention, including control of workplace exposures, medical surveillance, and treatment according to current asthma guidelines.

**What are the implications for public health practice?**

Prevention is best achieved through a multifaceted approach. It is essential to evaluate workers with new-onset or worsening asthma for relation to work exposures and to recognize work in cannabis production as potentially causative.

## Abstract

Multiple respiratory hazards have been identified in the cannabis cultivation and production industry, in which occupational asthma and work-related exacerbation of preexisting asthma have been reported. An employee working in a Massachusetts cannabis cultivation and processing facility experienced progressively worsening work-associated respiratory symptoms, which culminated in a fatal asthma attack in January 2022. This report represents findings of an Occupational Safety and Health Administration inspection, which included a worksite exposure assessment, coworker and next-of-kin interviews, medical record reviews, and collaboration with the Massachusetts Department of Public Health. Respiratory tract or skin symptoms were reported by four of 10 coworkers with similar job duties. Prevention is best achieved through a multifaceted approach, including controlling asthmagen exposures, such as cannabis dust, providing worker training, and conducting medical monitoring for occupational allergy. Evaluation of workers with new-onset or worsening asthma is essential, along with prompt diagnosis and medical management, which might include cessation of work and workers’ compensation when relation to work exposures is identified. It is important to recognize that work in cannabis production is potentially causative.

## Introduction

Studies in the cannabis cultivation and production industry have identified multiple respiratory hazards such as microbial and plant allergens and irritants, as well as chemicals, including pesticides, and allergens specific to the cannabis plant itself (*1*–*3*). Employees in some work areas are exposed to large quantities of ground cannabis. Respiratory and skin signs and symptoms, including asthma, allergic rhinitis, and urticaria, have been reported (*2*,*3*). Work-related asthma includes occupational asthma (new-onset asthma induced by sensitizers or irritants) and work-related exacerbation of preexisting asthma, worsened by work exposures (*4*). An employee working in a Massachusetts indoor cannabis facility experienced progressively worsening work-associated respiratory symptoms, which culminated in a fatal occupational asthma attack. This report provides information obtained in the public health investigation performed to determine the cause of this fatality and identify prevention options.

## Case Report

The employee, a woman aged 27 years, began work at an indoor cannabis cultivation and processing facility on May 20, 2021. She worked throughout the facility as a cycle counter, including in areas where the cannabis product was ground (Figure). In late July, she experienced onset of nausea, loss of taste and smell, earache, and cough, and her employer required her to obtain SARS-CoV-2 testing; the results of two tests were negative. Bilateral diffuse wheezing was noted when a physical examination was performed during the evaluation for the second test. The patient’s mother later reported that, although her daughter had no previous history of asthma, allergies, or skin rash, she had developed work-related runny nose, cough, and shortness of breath after 3–4 months of employment.

**FIGURE:**
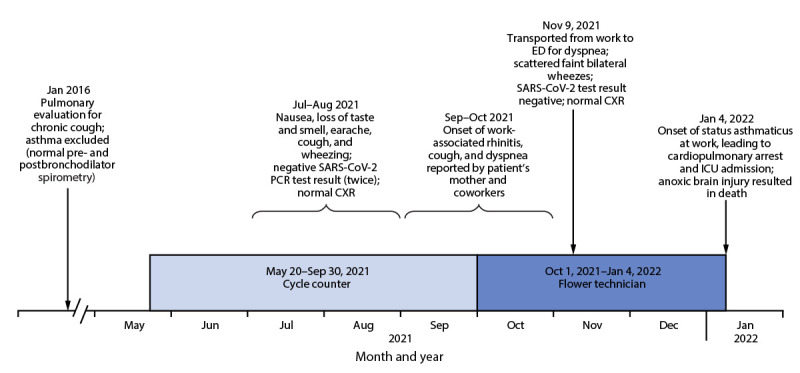
Timeline of work assignments,* onset of signs and symptoms, and events associated with fatal occupational asthma in a cannabis facility worker — Massachusetts, 2021–2022 **Abbreviations:** CXR = chest radiograph; ED = emergency department; ICU = intensive care unit; PCR = polymerase chain reaction. * Cycle counter’s responsibilities are counting packaged cannabis products throughout the facility, including in ground product areas; flower technician’s responsibilities are grinding cannabis flowers and making prerolls.

On October 1, the employee moved to flower production, which entailed grinding of cannabis flowers for approximately 15 minutes, three times per day, and preparing cannabis cigarettes (prerolls). These activities resulted in increased dust exposure. Dust from the grinder was collected by a shop vacuum; however, the vacuum had no high-efficiency particulate air (HEPA) filter, and visible dust escaped. Additional dust-generating processes included open handling of ground product (e.g., while transferring product from the grinder and filling prerolls). Other flower production coworkers reported that the employee’s cough increased, particularly when the grinder was on. Efforts to reduce her exposure included covering the grinder vacuum with plastic (the outside of which became visibly coated with ground cannabis) and moving her workstation outside the grinder room. She also used her own N95 respirator and wore company-required long sleeves and gloves while working.

On November 9, the employee became acutely dyspneic at work and was transported by emergency medical services (EMS) to a local emergency department (Figure). Enroute to the hospital, she received an albuterol nebulizer, and her dyspnea resolved. She reported that she did not have asthma but stated that she might be allergic to something at work because she had had a cough and runny nose for >1 month. Bilateral faint wheezes were noted, and she was prescribed a 5-day course of prednisone, cetirizine, and an albuterol inhaler; follow-up with a primary care physician was recommended. Her mother reported that the employee did not become short of breath at home, except when carrying a heavy load upstairs. She said that her daughter told her before her subsequent fatal asthma attack that the inhaler, which she used primarily at work, was nearly empty. This finding suggests that the employee had used most of the approximately 200 inhalations available in her inhaler over a period of approximately 2 months.

On January 4, 2022, the employee told a coworker that her shortness of breath had been getting progressively worse during the preceding 2 weeks. Later that day, while filling prerolls, she began sneezing, and her coughing increased. Despite repeated albuterol inhaler use, her dyspnea worsened, and EMS was called again. She suffered a cardiopulmonary arrest before EMS arrived, and her coworkers began resuscitation. She regained spontaneous circulation. However, she did not regain consciousness. Expiratory wheezing was noted. Anoxic brain death was diagnosed on January 7, 2022, and care was withdrawn. An autopsy was not performed.

## Public Health Investigation

The Massachusetts Department of Public Health investigation revealed that the employee had had a pulmonary evaluation in 2016 for chronic cough, which included pre- and postbronchodilator spirometry without a methacholine challenge (a bronchoprovocation test used to help diagnose asthma). The pulmonologist excluded asthma and implicated cigarette and marijuana smoking, gastroesophageal reflux disease, and rhinitis in the etiology of her cough symptoms. Her primary care physician had not seen the employee since 2015, and subsequently had not prescribed any allergy or asthma medication.

The Occupational Safety and Health Administration (OSHA) inspection included personal air sampling after the grinder was connected to a new shop vacuum with HEPA filtration. The 8-hour time-weighted average respirable dust concentration in air from the personal breathing zone of the grinder operator was 0.012 mg/m^3^, and for two nearby employees, was nondetectable; OSHA’s permissible exposure limit for respirable dust (particulates not otherwise regulated) is 5 mg/m^3^.* Additional 8-hour monitoring for endotoxin, a pro-inflammatory contaminant associated with gram-negative bacterial growth on organic materials such as cannabis flowers, revealed 27 endotoxin units per cubic meter of air (EU/m^3^) (grinder operator) and 1.8 and 1.9 EU/m^3^ (nearby employees); the Dutch Expert Committee on Occupational Safety 8-hour time weighted average recommendation is ≤90 EU/m^3^.^†^ A 15-minute personal air sample obtained from the personal breathing zone of the operator during active grinding was 14 EU/m^3^. OSHA interviewed one former and nine current flower production coworkers of the employee during February–April, 2022, four of whom reported work-related respiratory tract or skin signs and symptoms; symptoms in the former employee suggested occupational asthma, because, although he had a past history of asthma, he had not required a bronchodilator inhaler since adolescence. This activity was reviewed by CDC, deemed not research, and was conducted consistent with applicable federal law and CDC policy.^§^

## Discussion

Cannabis industry employees are exposed to large quantities of ground product in some work areas, such as flower grinding and preroll production. Asthma, allergic rhinitis, and urticaria have been reported among cannabis production workers (*2*,*3*). Several allergens have been identified, and irritants are present as well (*1*–*3*). Work-related asthma includes occupational asthma (i.e., new-onset asthma induced by sensitizers or irritants) and work-exacerbated asthma (i.e., preexisting asthma worsened by work exposures) (*4*). In this case, absence of a history of asthma and the temporal relationship between work exposure and asthma signs and symptoms are consistent with a diagnosis of occupational asthma. Airborne respirable dust and endotoxin levels below occupational exposure limits do not exclude a sufficient level of airborne allergen to trigger asthma and other allergic symptoms.

Enhanced surveillance for work-related asthma in the state of Washington identified seven asthma cases among employees in indoor cannabis production facilities (*5*). Three employees with work-exacerbated asthma discontinued cannabis employment; one with occupational asthma was symptomatic in two different cannabis facilities separated by a 2-year asymptomatic period while unexposed.

In a study of employees at an indoor Washington cannabis production facility, 13 of 31 employees had symptoms suggestive of asthma (i.e., presence of either an attack of shortness of breath, an attack of asthma, or the use of asthma medication) (*6*). Among 10 employees with occupational allergy symptoms, seven had abnormal spirometry, and five had skin prick testing consistent with cannabis sensitization. Five employees had abnormal or borderline fractional exhaled nitrogen oxide testing, which is used as a marker of airway inflammation in asthma management; results increased significantly across the work week, indicating an increase in airway inflammation.

Fatal asthma can occur even with disease that is considered mild; disparities in income, education, and access to health care are risk factors associated with death (*7*). Work-related asthma has also been associated with poorer asthma control (*8*). Additional risk factors for the deceased employee in this case report include the emergency department visit, recent use of oral glucocorticoids, increased dyspnea and bronchodilator inhaler use without inhaled glucocorticoids, continued exposure, and lack of a provider with expertise in occupational allergies (*7*,*9*).

Occupational asthma is generally associated with a latency period of months to years between first exposure and symptoms (*10*). For example, fatal occupational asthma related to exposure to powdered shark cartilage was reported 16 months after exposure onset (*10*). Although latency from this employee’s first occupational cannabis exposure to symptom onset was short, latency from first exposure was longer because of personal cannabis use. Cross-sensitivity between cannabis and plant allergens might also have predisposed this employee to cannabis sensitization (*3*).

### Limitations

The findings in this report are subject to at least three limitations. First, although the employee’s course is consistent with fatal asthma triggered by cannabis allergy, this finding was not evaluated by skin testing or specific immunoglobulin E tests. Second, airborne cannabis allergen levels could not be assessed. Finally, as in many occupational fatality cases, investigators were not able to speak with the employee, requiring details to be obtained from other sources such as medical records and interviews with coworkers and next-of-kin.

### Implications for Public Health Practice

Providers and public health professionals would benefit from additional research into prevalence and risk factors for cannabis-related occupational allergies. Development and implementation of strategies to protect workers are critical in this rapidly expanding industry. Measures to protect employees might include determination and control of exposures, training of employees and facility managers, correct use of personal protective equipment, and medical management of employees with work-related symptoms, which might require cessation of work and workers’ compensation (Box). It is important to recognize that work in cannabis production is a risk for occupational allergies.

BOX **Measures for protecting cannabis industry employees from occupational hazards — United States, 2023**

Exposure Assessment*^,†^

- Qualitative assessment to identify areas and processes of highest potential dust exposure
- Quantitative assessment of airborne levels as needed to assist in evaluating controls for dust and other exposures

**Environmental Exposure Controls**

- Equipment controls (e.g., exhaust ventilation for cannabis grinder) to mitigate risk from dust-producing processes
- Work procedures to reduce airborne dust (e.g., high-efficiency particulate air–filtered vacuuming rather than dry sweeping)

**Personal Protective Equipment**

- In dusty settings, personal protective equipment for skin (e.g., gloves, long sleeves, or sleeve guards), eyes (e.g., safety glasses or goggles) and respiratory protection (e.g., an N95 particulate respirator) as needed
- However, personal protective equipment might not be effective for persons with signs and symptoms of work-related allergies

**Employee Training**

- To identify potential job hazards
- To recognize signs and symptoms of occupational allergy (e.g., rhinitis, conjunctivitis, asthma, and urticaria; particularly if new-onset or worse at work)
- To seek prompt medical evaluation for signs and symptoms of occupational allergy
- To use work processes that minimize exposures*
- To use and maintain personal protective equipment

**Medical Surveillance**

- Directed by a health care provider with expertise in occupational allergy and asthma
- Focused on early detection of signs and symptoms of occupational allergy
- Aggregated analysis of all workers’ results to identify exposures and jobs that result in highest risk for allergic sensitization and disease

**Medical Management Options and Workers’ Compensation**

- Workplace restrictions for sensitized persons, recognizing that complete cessation of exposure rather than exposure reduction might be necessary
- Recognition of work-related allergic sensitization potential in cannabis industry employees for workers’ compensation claims and regulations

**Examples of Current Research Gaps**

- Development of exposure assessment methods and exposure controls to facilitate effective prevention of occupational allergic disease
- Assessment of prevalence and risk factors for occupational allergy and disease in cannabis workers
- Development of reliable, clinically available diagnostic tests for cannabis sensitization

* https://stacks.cdc.gov/view/cdc/91903

^†^ https://www.researchgate.net/publication/369800248_The_Emerging_Spectrum_of_Respiratory_Diseases_in_the_US_Cannabis_Industry

## Footnotes

*
https://www.osha.gov/laws-regs/regulations/standardnumber/1910/1910.1000TABLEZ1

† A recommended short-term exposure limit for endotoxins has not been established. Importantly, airborne respirable dust and endotoxin levels below occupational exposure limits do not exclude work-related triggers of asthma and other allergic signs and symptoms (e.g., cannabis allergens). https://www.healthcouncil.nl/documents/advisory-reports/2010/07/15/endotoxins-health-based-recommended-occupational-exposure-limit

§ 45 C.F.R. part 46.102(l)(2), 21 C.F.R. part 56; 42 U.S.C. Sect. 241(d); 5 U.S.C. Sect. 552a; 44 U.S.C. Sect. 3501 et seq.

## References

[1] Couch JR, Grimes GR, Green BJ, Wiegand DM, King B, Methner MM. Review of NIOSH cannabis-related health hazard evaluations and research. Ann Work Expo Health 2020;64:693–704. 10.1093/annweh/wxaa013 32053725 PMC7416426

[2] Decuyper II, Green BJ, Sussman GL, Occupational allergies to cannabis. J Allergy Clin Immunol Pract 2020;8:3331–8. 10.1016/j.jaip.2020.09.003 33161961 PMC7837257

[3] Sack C, Simpson C, Pacheco K. The emerging spectrum of respiratory diseases in the U.S. cannabis industry. Semin Respir Crit Care Med 2023;44:405–14. 10.1055/s-0043-1766116 37015286 PMC10449032

[4] Tarlo SM, Balmes J, Balkissoon R, Diagnosis and management of work-related asthma: American College of Chest Physicians consensus statement. Chest 2008;134(Suppl):1S–41S. 10.1378/chest.08-0201 18779187

[5] Reeb-Whitaker C, LaSee CR, Bonauto DK. Surveillance of work-related asthma including the emergence of a cannabis-associated case series in Washington state. J Asthma 2022;59:1537–47. 10.1080/02770903.2021.1955379 34288786

[6] Sack C, Ghodsian N, Jansen K, Silvey B, Simpson CD. Allergic and respiratory symptoms in employees of indoor *Cannabis* grow facilities. Ann Work Expo Health 2020;64:754–64. 10.1093/annweh/wxaa050 32459852 PMC7407609

[7] Madison JM, Irwin RS. Identifying patients at risk for fatal asthma. Waltham, MA: UpToDate; 2023. Accessed April 7, 2023. https://www.uptodate.com/contents/identifying-patients-at-risk-for-fatal-asthma

[8] Mazurek JM, Henneberger PK. Use of population data for assessing trends in work-related asthma mortality. Curr Opin Allergy Clin Immunol 2019;19:98–104. 10.1097/ACI.0000000000000508 30601151 PMC6892609

[9] National Heart, Lung, and Blood Institute. Expert panel report 3: guidelines for the diagnosis and management of asthma. Bethesda, MD: US Department of Health and Human Services, National Institutes of Health; 2007. https://www.nhlbi.nih.gov/sites/default/files/media/docs/EPR-3_Asthma_Full_Report_2007.pdf

[10] Ortega HG, Kreiss K, Schill DP, Weissman DN. Fatal asthma from powdering shark cartilage and review of fatal occupational asthma literature. Am J Ind Med 2002;42:50–4. 10.1002/ajim.10088 12111690

